# PMMA interlayer-modulated memory effects by space charge polarization in resistive switching based on CuSCN-nanopyramids/ZnO-nanorods p-n heterojunction

**DOI:** 10.1038/srep17859

**Published:** 2015-12-09

**Authors:** Baochang Cheng, Jie Zhao, Li Xiao, Qiangsheng Cai, Rui Guo, Yanhe Xiao, Shuijin Lei

**Affiliations:** 1 School of Materials Science and Engineering, Nanchang University, Jiangxi 330031, P. R. China; 2 Nanoscale Science and Technology Laboratory, Institute for Advanced Study, Nanchang University, Jiangxi 330031, P. R. China

## Abstract

Resistive switching (RS) devices are commonly believed as a promising candidate for next generation nonvolatile resistance random access memory. Here, polymethylmethacrylate (PMMA) interlayer was introduced at the heterointerface of p-CuSCN hollow nanopyramid arrays and n-ZnO nanorod arrays, resulting in a typical bipolar RS behavior. We propose the mechanism of nanostructure trap-induced space charge polarization modulated by PMMA interlayer. At low reverse bias, PMMA insulator can block charges through the heterointerface, and 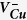 and 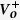 trapped states are respectively created on both sides of PMMA, resulting in a high resistance state (HRS) due to wider depletion region. At high reverse bias, however, electrons and holes can cross PMMA interlayer by Fowler-Nordeim tunneling due to a massive tilt of energy band, and then inject into the traps of ZnO and CuSCN, respectively. 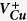 and 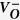 trapped states are created, resulting in the formation of degenerate semiconductors on both sides of PMMA. Therefore, quantum tunneling and space charge polarization lead to a low resistance state (LRS). At relatively high forward bias, subsequently, the trapped states of 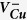 and 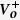 are recreated due to the opposite injection of charges, resulting in a recovery of HRS. The introduction of insulating interlayer at heterointerface, point a way to develop next-generation nonvolatile memories.

Digital type resistive switching (RS), showing discrete transition between low resistance state (LRS) and high resistance state (HRS) with a large difference of resistance between them, has been pursued for reliable memory operation. Resistive random access memory (RRAM), based on RS effect, is considered as one of the most competitive candidates for next-generation nonvolatile memories. Especially for the write-once-read-many-times (WORM) resistive devices, they have attracted a great deal of interest because of their fast switching speed, low power consumption, high-density integration and extensive applications in rapid archival storage equipment^1^,^2^. Although RS behaviors have been widely investigated in various materials, many aspects of the microscopic switching mechanism remain unclear. Although the most believable explanation is the formation and rupture of conductive filaments in insulators, the physicochemical processes at the nanoscale are still controversial^3^,^4^,^5^,^6^. Other plausible mechanisms, such as field-assisted drift/diffusion of charged ions, trap-controlled space-charge-limited current, Frenkel−Poole emission, Schottky barrier, and mass transfer have been extensively proposed^7^,^8^,^9^,^10^,^11^.

Nowadays, the prospect of promising applications of nonvolatile memory devices utilizing hybrid inorganic/organic composites containing inorganic nanostructures has became a promising type of RS device. Such nanomaterials have large surface and interface area, and therefore they can provide effectively trapping and hopping sites for charge transport and act as reservoir and supplier of defects, resulting in a dramatic change in the physical properties. It is studied numerously for its potential application in nonvolatile memory devices, such as low production cost, printability, simple fabrication and good scalability, and the metal oxide based heterojunctions can be prepared on various types of substrates, thus providing flexible processing options^11^,^12^,^13^,^14^,^15^. The RS device is controlled by an external electric field with opposite polarity and can achieve the purpose with the repeated resistance variation between HRS and LRS. The two levels of different conductivities of devices can be switched under suitable voltages, enabling the ability to the data storage.

Zinc oxide (ZnO) is a direct bandgap semiconductor with an energy gap of about 3.37 eV and a large exciton binding energy of 60 meV at room temperature^16^,^17^. Due to the peculiar physical properties from confined dimensions, nanostructured ZnO has been extensively applied in optoelectronic and electronic devices, such as solar cells^18^,^19^,^20^,^21^,^22^, light emitting devices^23^,^24^, gas sensors^25^, and photodetectors^26^. Recently, nanostructured ZnO has attracted much attention as potential candidate for RS memory device as well^27^,^28^,^29^,^30^,^31^,^32^,^33^,^34^,^35^. For one-dimensional (1D) nanostructure ZnO-based RS memories, they generally have extreme properties with ultralow leakage current in HRS, large memory window, and low switching voltage^32^,^33^,^34^,^35^. So far, polymers have been applied to the active layer in the RS devices^13^,^14^,^15^, but it is rarely to use the polymers to connect the two types of semiconductors as RS devices. PMMA is a polar material with low nonlinear refractive index, good plasticity and large dielectric constant, and therefore it has a potential application in optoelectronics and electronics^36^. The p-CuSCN is a promising hole-transporting material due to its reasonable hole conductivity (≥5 × 10^−4^ S/cm), chemical stability and transparence in visible light spectrum range^37^. It is expected to form p-n heterojunction diode when n-type ZnO nanorods directly contact with p-type CuSCN films^38^,^39^,^40^.

Although RS based on p-n diodes have been prepared, the oxide-based diodes often show problems due to somewhat poor electrical properties of the p-type oxide and also due to possible fabrication damage in the p–n junction, so that they may not properly play as a dynamic switching device^41^. So far, the origins of RS behaviors in diode-based WORM remain unclear. In addition, nanodevices based on single nanostructures are not suitable for semiconductor manufacturing purposes, as it is difficult to control the electronic properties, growth, and alignment of individual nanostructures on an industrially reliable scale. For practical device application, however, it is necessary to fabricate a large-area and well-ordered parallel array of periodically spaced and identical-size nanostructures for wafer-scale integration into an active parallel nanoarchitecture in real-world devices^42^. In this paper, in order to modulate and gain insight into the diode-based RS mechanism, a sandwich-like p-i-n heterostructure wafer was constructed using ZnO nanorod arrays, PMMA and CuSCN hollow nanopyramid arrays as n, insulator and p layer, respectively. Simultaneously, the voltage bias was applied to the two ends of ITO electrodes during the electrical measurements. The results reveal that it only shows a diode feature with relatively low rectification ratio for the nanostructure-based film device of CuSCN/ZnO p-n heterojunction, whereas the introduction of PMMA interlayer results in a significant bipolar RS effect for the CuSCN/PMMA/ZnO p-i-n heterojunction device; moreover, a large hysteresis behavior indicates a good nonvolatile data storage capability.

## Results

**Figure 1:**
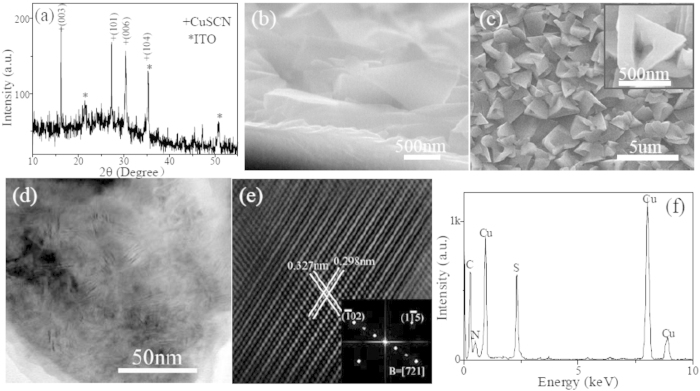
Structural characterization of as-prepared CuSCN hollow pyramid arrays. (**a**) XRD pattern. FESEM images of cross-sectional (**b**) and top-sectional (**c**) view. The inset in (**c**) corresponds to higher magnification FESEM image, showing a hollow pyramid structure. (**d**) Bright-field TEM image, showing the presence of quantities of defects. (**e**) HRTEM image of typical defect structure in CuSCN. The inset corresponds to FFT analysis. (**f**) EDS pattern.

The XRD pattern of electrodeposited CuSCN thin film is shown in Fig. 1a. Besides ITO diffraction peaks, originating from the substrate, other reflection peaks can be indexed to a rhombohedral structure β−CuSCN, which is in good agreement with the report data for CuSCN (JCPDS Card File No. 00–029–0581). No characteristic peaks from other crystalline forms are detected within the XRD detection limit. From the cross-sectional and top-sectional view SEM images, respectively shown in the Fig. 1b,c, it can be obviously seen that the electrodeposited CuSCN film is uniform with a thickness of approximately 2 μm, and moreover the grains grow in regular hollow pyramid shape with the size of about 400 nm. As seen from TEM image in Fig. 1d, quantities of structural defects exist in CuSCN lattice. HRTEM image and corresponding fast Fourier transform (FFT) analysis, as shown in Fig. 1e, further verify that defects are mainly composed of supperlattices and stacking faults. The compositional analysis was performed by the energy dispersive X-ray spectroscopy (EDS) equipped in HRTEM, as shown in Fig. 1f, indicating that the sample is composed of Cu, S, C and N.

**Figure 2:**
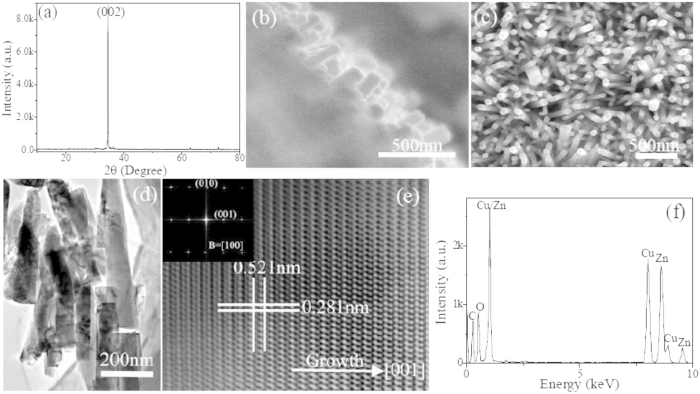
Structural characterization of as-prepared ZnO nanorod arrays. (**a**) XRD pattern. FESEM images of cross-sectional (**b**) and top-sectional (**c**) view. (**d**) Bright-field TEM image. (**e**) HRTEM image. The inset corresponds to FFT analysis. (**f**) EDS pattern.

The XRD pattern of as-grown ZnO nanostructures is presented in Fig. 2a. It can be clearly seen that a single prominent diffraction peak near the diffraction angle of 34°, associated with the (002) plane of hexagonal wurtzite structure ZnO, appears in the pattern, which demonstrates that the nanostructures are preferentially oriented along the c-axis. Low-magnification FESEM observation displays the cross-sectional SEM micrographs (Fig. 2b) of ZnO, and it confirms clearly that the as-prepared product is composed of a great quantity of rod-like nanostructures. As seen from the top-sectional view of ZnO (Fig. 2c), highly dense uniform growth of nanostructures is oriented and almost vertical to the substrate with the diameter of around 100 nm. As seen from TEM bright-field and high-resolution images, and corresponding FFT analysis, shown in Fig. 2d,e, the growth of ZnO nanorods is along c-axis. The EDS compositional analysis reveals that the nanorods are composed of Zn and O besides the Cu and C from carbon-coated TEM grid.

**Figure 3:**
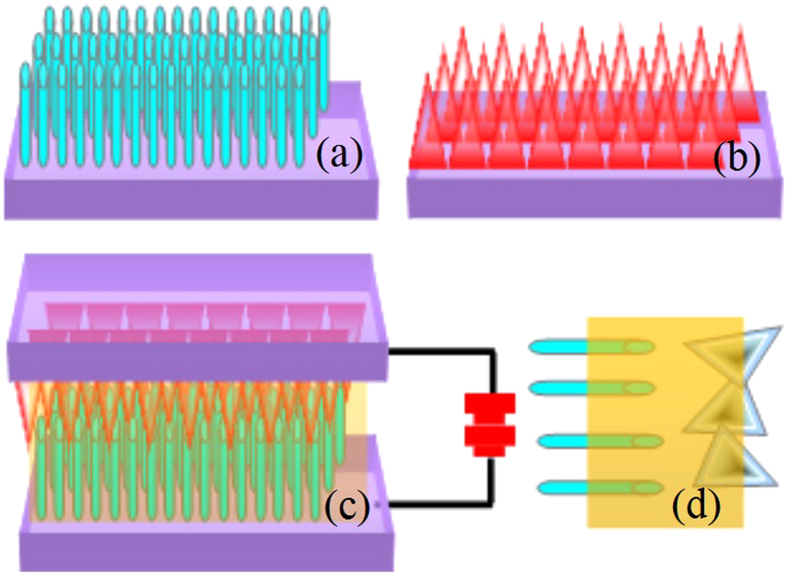
Schematic diagrams of RS device structure. (**a**) ZnO nanorod arrays grown vertically on ITO glass. (**b**) CuSCN hollow pyramid arrays grown on ITO glass. (**c**) CuSCN/PMMA/ZnO RS device. (**d**) A magnification of CuSCN/PMMA/ZnO contact region, indicating a p-i-n structure.

Commercial ITO glass, which is degenerate n-type semiconductor, is employed as the substrate of CuSCN and ZnO growth and the transparent electrode. We fabricated an about 1×l cm testing cell with the CuSCN/PMMA/ZnO to investigate the electrical property of p-i-n devices. The corresponding schematic diagram is shown in Fig. 3. It is revealed in Fig. 3a that the ZnO nanorods on the ITO obtained by the hydrothermal method are regular and vertical, and the CuSCN film on the ITO as Fig. 3b shows is obtained by the electrodeposition method. As presented in Fig. 3c, the ZnO is employed as n-type and the CuSCN is employed as p-type, and then PMMA is added between them to separate the CuSCN and ZnO. In the enlarged picture of internal structure of the RS (Fig. 3d), the CuSCN and ZnO are not connected. As a consequence, a typical p-i-n structure device is fabricated.

**Figure 4:**
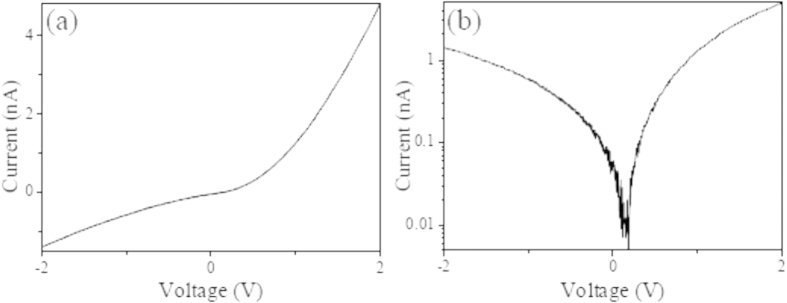
Typical I-V characteristics of CuSCN/ZnO p-n heterojuction device. (**a**) Linear scale. (**b**) Semi-logarithmic scale.

The representative current-voltage (I-V) characteristics of the CuSCN/ZnO p-n heterojunction device are described both on a linear scale and a semi-logarithmic scale, as shown in Fig. 4. All the bias voltages were applied to the two end ITO electrodes of device. As seen from the linear scale (Fig. 4a) and the semi-logarithmic scale (Fig. 4b) of I-V curves, the device displays a conductive state when a forward bias voltage is applied to the device, and however it reveals a relatively high leakage current when a reverse bias voltage is applied, implying a relatively poor rectifier characteristics with rectification ratio of about 3.5 at ± 2 V bias voltage. In addition, the p-n devices do not display any observable switching effect.

**Figure 5:**
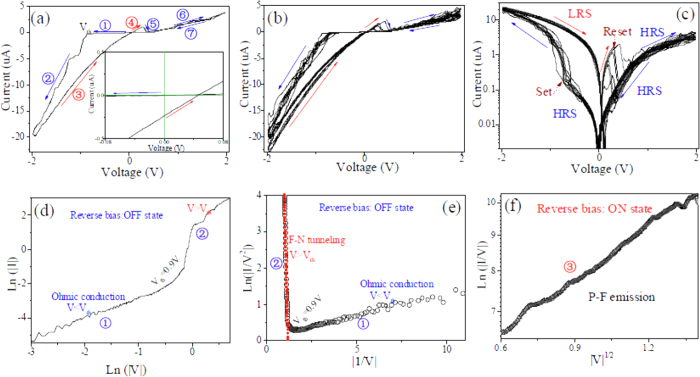
Representative I-V characteristics of CuSCN/PMMA/ZnO p-i-n device. (**a**) One cycle on linear scale, implying a bipolar RS feature. The numbered arrows (1–7) indicate the direction of sweep voltages. The inset corresponds to the expanded view of I-V curve near zero voltage, revealing that I-V curve deviates from zero point upon sweeping from −2 to 2 V. (**b**) 7 consecutive cycles in linear scale. (**c**) Corresponding semi-logarithmic scale in (**c**). (**d**) Fitted I-V characteristics of OFF state by Ohmic conduction mechanism in the range of 0 to −2 V. (**e**) Fitted I-V characteristics of OFF state by Fowler-Nordeim tunneling mechanism in the range of 0 to −2 V. (**f**) Fitted I-V characteristics of ON state by Poole–Frenkel conduction mechanism in the range of −2 to 0 V.

For the CuSCN/PMMA/ZnO p-i-n devices, the I-V curves were swept by applying a bias voltage of 2 V to the ITO electrodes with the variations of voltages across the heterojunction device in a cyclic manner. As seen from the linear scale (Fig. 5a) and the semi-logarithmic scale (Fig. 5b), the I-V characteristics show a significantly difference compared with the CuSCN/ZnO p-n device. At forward bias, the current of p-i-n heterojunction device is higher, which indicates that more conductive channels are formed since it is easy to adsorb each other between nanostructured CuSCN and ZnO in MMA solvent. At low reverse bias, however, the leakage current decreases dramatically and the device is non-conductive. With increasing reverse biased voltage, more interestingly, the leakage current of as-prepared p-i-n device increases abruptly and the SET switching can start to transfer from HRS to LRS at about −0.9 V (threshold voltage, V_th_). Then the device remains in the LRS when the applied voltage is decreased from −2 to about 0.3 V, and moreover the I-V curve deviates from zero point. In addition, the I-V curves show relatively large hysteresis loops at negative bias, indicative of nonvolatile data storage capability. As the voltage is subsequently increased toward positive values, the current suddenly begin to drop at about 0.3 V, showing an apparent negative differential resistance (NDR) feature, and the device current switches back to the high-resistance OFF state (RESET). When the bias voltage exceeds certain negative voltage of about −0.9 V, the device is returned to the LRS state, and subsequently it is switched back to the HRS state upon increasing the positive bias voltage to about 0.3 V. This phenomenon implies that the p-i-n heterojunction-based RS cell has a relatively low operation voltage, and forms ON state at around −0.9 V and OFF states at about 0.3 V, respectively; moreover, these results strongly indicate that it is beneficial for the introduction of PMMA interlayer at the CuSCN/ZnO interface to form bipolar RS.

**Figure 6:**
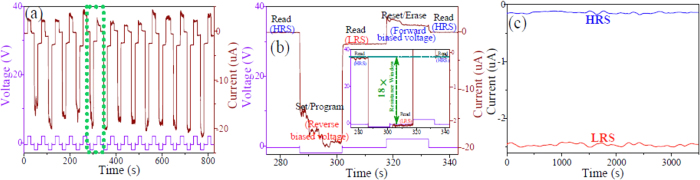
Write/read access of RS cell as a memory. (**a**) A programmable pulse voltage response. −2, 2 and −0.5 V is served as writing, erasing, and reading voltage, respectively. The wine curves correspond to respondent current, and the violet curves correspond to applied voltage. (**b**) An enlargement of a green frame in (**a**) and the inset corresponds to a higher magnification for LRS and HRS current, showing a detail reading-writing-reading-erasing-reading process and a resistance window of about 18 times. (**c**) Stability of cell resistance at room temperature in both the LRS and HRS at reading voltage of −0.5 V.

The CuSCN/PMMA/ZnO devices exhibit bipolar RS behavior upon being applied a cyclic sweep voltage. More interestingly, however, the resistivity of device can be restored to pristine HRS after being applied a relatively high forward bias voltage, namely, reset process. Therefore, the RS cell can be used as a memory. A single selected cell was tested under the DC sweeping mode by conducting a series of successive programming/erasing cycles, and the results are illustrated in Fig. 6. It is observed for thirteen repeated programming-reading-erasing-reading cycles. It can be seen that the information can be set/programmed by applying a relatively high reverse bias voltage of −2 V, read by applying a relatively low reverse bias voltage of −0.5 V, and reset/erased by applying a relatively high forward bias voltage of 2 V. After applying reverse and forward bias voltages of ±2 V, the device shows about 3.5 MΩ resistance difference and 18× resistance window in HRS and LRS under reading out at −0.5 V. Additionally, the current can rapidly respond to the variation of bias voltage, indicating that the devices can be set/reset in a short time. Figure 6c shows the variation of the resistance with time both at HRS and LRS. As can be seen, the variation of LRS and HRS resistance after 3500 seconds is found to be very little at the reading voltage of −0.5 V, confirming the nonvolatile behavior of the device.

## Discussion

**Figure 7:**
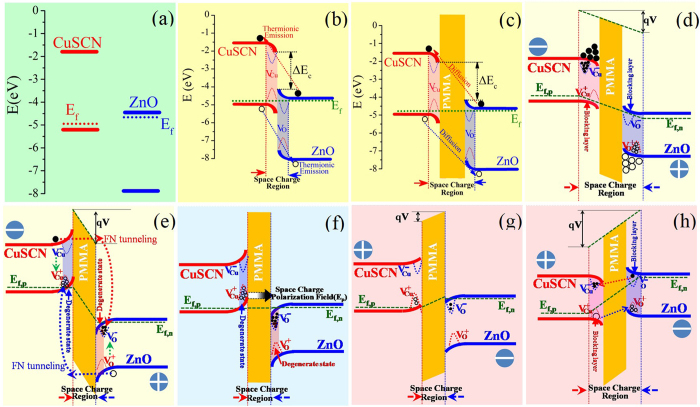
Schematic illustrations of charge distribution and corresponding energy band structure at different bias voltages. (**a**) Before contact of p-CuSCN and n-ZnO. (**b**) Direct contact of p-CuSCN and n-ZnO without bias, revealing the presence of surface states and the thermionic emission dominated conduction mechanism. (**c**) CuSCN/PMMA/ZnO p-i-n structure without bias. (**d**) A low reverse-biased CuSCN/PMMA/ZnO structure at OFF state (V<V_th_). Electrons and holes are respectively accumulated within CuSCN and ZnO near PMMA interlayer, resulting in wider blocking layers due to the formation of 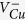 and 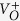. (**e**) A high reverse-biased CuSCN/PMMA/ZnO structure at ON state (V >V_th_). Charges cross freely PMMA by FN tunneling, the degenerate semiconductors is formed both on sides of PMMA due to the formation of 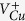 and 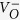. (**f**) Without bias after applying high reverse bias, revealing the presence of space charge polarization and quantum tunneling effects. (**g**) A low forward-biased voltage at reset point. The CB of ZnO is close to the VB of CuSCN and quantum tunneling disappears, resulting in the recovery of HRS. (**h**) A high forward-biased voltage at OFF state. Charges cross the PMMA interlayer from opposite direction, resulting in the re-formation of 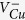 and 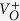.

To better clarify the conduction and switching dynamics of the memory cell, the energy band diagrams of the heterojunctions based on Anderson’s model, given in Fig. 7. The band gap and electron affinity values for ZnO and CuSCN are E_g,ZnO_ = 3.37 eV, χ_ZnO_ = 4.35 eV^43^, and E_g,CuSCN_ = 3.6 eV, χ_CuSCN_ = 1.5 eV^44^, respectively. As seen from the schematic diagram of energy level structure (Fig. 7a), the conduction band (CB) of ZnO is close to the valence band (VB) of CuSCN in energy. Especially for light doping, their Fermi level is much closer. When they contact directly, therefore, the barrier height (E_f,ZnO_-E_f,CuSCN_) of p-n heterojunction is relatively low. Owing to the presence of surface states in nanostructures, however, the depletion region (space charge region) still exists in CuSCN and ZnO heterointerface, and the thermionic emission is dominantly mechanism for the charge transport in heterointerface due to narrow depletion region, as illustrated in Fig. 7b. At reverse bias, it is easy for charges to cross the heterointerface by direct tunneling. Therefore, the rectifying characteristics of CuSCN/ZnO p-n heterojunction are relatively poor due to a relatively large reverse leakage current.

For 1D nanostructured ZnO, quantities of surface states exist^45^,^46^,^47^. Moreover, abundant structural defects exist in nanostructured CuSCN lattice as well, verified by HRTEM analysis. These defects can serve as trap centers and capture injected charges. When the PMMA insulator layer is introduced at the interface of CuSCN/ZnO heterojunction, it can serve as charge-blocking layer and decrease the recombination between trapped electrons and hole due to a relatively large energy gap, resulting in the respective accumulation of electrons and holes on both sides of the PMMA at low reverse bias, as illustrated in Fig. 7c. Then, electrons and holes can be captured and trapped in the defects of CuSCN and ZnO near the PMMA, respectively, and correspondingly 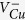 and 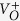 are formed, resulting in much wider space charge regions. Therefore, the reverse leakage current can be reduced effectively. For V<V_th_ region, a linear relationship can be observed between ln(|I|) versus ln|V|, as shown in Fig. 5f. In this case, the applied voltage is less than the barrier height of PMMA, charges tunnel through a trapezoidal barrier^48^, i.e., direct tunneling occurs. This corresponds to the HRS of devices.

With increasing reverse bias, the PMMA bands bend gradually to become triangular. When the applied voltage exceeds the barrier height, charges tunnel through the triangular barrier, which is analogous with field emission and Fowler–Nordheim (FN) tunneling occurs^49^,^50^. Electrons and holes can cross PMMA freely, and then inject into the defects of ZnO and CuSCN near the PMMA, respectively. The 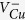 and 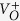 can recombine with the injected holes and electrons, respectively, and then empty. Moreover, the excess injection holes and electrons result in the fill of hole and electron traps in CuSCN and ZnO, respectively, and then 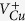 and 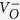 are formed in CuSCN and ZnO near the PMMA. Thus anti-blocking layer is formed, which is beneficial for charge transport. Charge filling leads to space charge polarization effect. For V>V_th_ region, a linear decrease relation of ln(|I/V^2^|) versus |1/V| can be found, as illustrated in Fig. 5e. This indicates a typical FN tunneling. With decreasing reverse bias voltage from −2 to 0, a linear decrease relation of ln(|I/V|) versus 1/V^2^ can be achieved, as shown in Fig. 5h. It corresponds to a Poole–Frenkel (P-F) conduction mechanism, referred to electric-field-enhanced thermal emission from a trapped state into a continuum of electronic states^43^. This further verifies the presence of trapped states in nanostructures.

In the vicinity of PMMA, the electron traps of ZnO are filled up, resulting in the shift of E_f_ toward ZnO CB. When the E_f_ is above the CB level, the degenerate semiconductor is formed. Simultaneously, the degenerate CuSCN semiconductor is also formed near the PMMA due to the filling of holes. Moreover, the energy level of ZnO CB is lower than that of CuSCN VB. Quantities of quantum states with the same energy exist on both sides of the PMMA, resulting in a tremendous increase of quantum tunneling probability. As a consequence, both of the space charge polarization effect from charge localization and the quantum tunneling effect from degenerate semiconductor lead to a LRS. The introduction of PMMA decreases the recombination of trapped charges within ZnO and CuSCN, resulting in a stable space charge polarization behavior. The polarized field creates an additional conductive path. After applying a high reverse bias, therefore, the I-V curve deviates from zero point when the voltage sweeps from negative to positive bias, as illustrated in the inset of Fig. 5a.

When a low forward bias is applied, LRS is still dominant due to the presence of space charge polarization and quantum tunneling effect. At high forward bias voltage, however, electrons and holes can freely cross PMMA insulator by direct tunneling, and then inject into CuSCN and ZnO, respectively. The trapped holes in CuSCN and electrons in ZnO can recombine with the injected electrons and holes, respectively. The CB edge of ZnO upshifts, while the VB edge of CuSCN downshifts. When energy level of ZnO CB is higher than that of CuSCN VB, the quantum tunneling disappears, and meanwhile the space charge polarization effect disappears as well, resulting in the recovery of HRS and the appearance of NDR behavior, namely resetting or erasing process. The noticeable NDR behavior strongly indicates that the transport mechanism is closely related to the quantum tunneling between ZnO CB and CuSCN VB. With further increasing forward bias, the excess injection electrons and holes can be captured and trapped again. Thus, 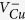 and 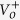 trapped states are re-created, and depletion regions are re-formed. It is due to the filling and emptying of electron and hole trap states that causes RS memory effects. CuSCN/PMMA/ZnO nanostructured devices, served as nonvolatile RRAM, can show a promising programming and erasing effect.

In summary, the nonvolatile RS memory nanodevices based on CuSCN/PMMA/ZnO p-i-n heterojuction were constructed. Upon PMMA interlayer being introduced at the interface of heterojunction consisting of p-type CuSCN hollow nanopyramid arrays and n-type ZnO nanorod arrays, the devices exhibit the reliable bipolar RS behavior with LRS at relatively high reverse bias and HRS at relatively high forward bias. The PMMA interlayer blocks the recombination between trapped electrons and holes at low reverse bias, and correspondingly increases the width of depletion region and forms HRS. At high reverse bias, however, the massive tilt of energy band induces the FN tunneling, which can make quantities of electrons and holes injected into ZnO and CuSCN, respectively, resulting in the formation of 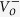 and 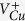 trapped states. The trap filling leads in the formation of degenerate semiconductors on both sides of the PMMA. As a consequence, the quantum tunneling and space charge polarization effects create LRS. At relatively high forward bias, subsequently, 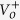 and 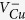 trapped states can be re-created, and ZnO and CuSCN, close to the PMMA, are back to non-degenerate semiconductors due to the charge injection from the opposite direction. As a result, the devices are switched to HRS accompanied with a NDR. It is the repeated formation and disappearance of quantum tunneling and space charge polarization due to the charge injection into/from abundant defect-related traps of nanostructures that nonvolatile RS memory can be obtained effectively. The WORM memory devices with excellent RS properties, enhanced by the introduction of insulating interlayer at the interface of p-n heterojunction, point a way to the development of next-generation nonvolatile memory.

## Methods

### Growth of n-type ZnO nanorod arrays

The synthesis of n-type ZnO nanorods on indium tin oxide (ITO) substrate involves the two-step process of seed generation and chemical-bath method. Firstly, the glycol ether solution with 0.75 M zinc acetate and 0.75 M ethanolamine was used as the seed solution, and then the seed layer on the ITO was obtained by spin-coating method under 4500 rpm for 30 s. This was followed by annealing for 1 h in air atmosphere at 500 °C. After the ZnO-seed layer was deposited, ZnO nanorod arrays were formed in an aqueous solution of zinc nitrate 0.05 M and hexamethylenetetramine 0.05 M at 95 °C for 4 h, the products were rinsed with deionized water and air-dried.

### Electrodeposition of p-type CuSCN nanopyramids

For the fabrication of p-CuSCN film, it was electrodeposited on the ITO using a three-electrode system with a graphite counter electrode and an Hg/HgCl reference electrode at room temperature. A stable weak basic aqueous (pH~9) solution consisted of 0.01 M CuSO_4_·5H_2_O, 0.05 M KSCN and 0.10 M monoethanolamine (MEA, C_2_H_7_NO). The peak voltage of the pulse voltage, move up voltage, rate and reaction time during deposition were 30 mV, 30 mV, 1 KHz and 10 min, respectively. The products were rinsed with deionized water and air-dried.

### Fabrication of p-i-n heterostructure devices

In order to produce the RS device, MMA solvent was introduced into the interface between CuSCN and ZnO, and then they were naturally bonded together under gravity and Van der Waals force. Finally, the RS device was obtained after the MMA was solidified into PMMA at 180 °C.

### Characterization

The morphology and microstructure of as-synthesized CuSCN and ZnO samples were characterized by X-ray diffraction (XRD, RIGAKU D/max-3b), field-emission scanning electron microscopy (FE-SEM, FEI Quanta 200F) and high-resolution transmission electron microscopy (HRTEM, JEOL JEM-2100). Static current-voltage (I-V) measurements for confirming RS were carried out using a synthesized function generator (Stanford Research System Model DS345) and a low-noise current preamplifier (Stanford Research System Model SR570). During the measurements in voltage sweeping mode, the bias was defined as positive when the current flowed from the CuSCN to ZnO, and the negative bias was defined by the opposite direction.

## Additional Information

**How to cite this article**: Cheng, B. *et al.* PMMA interlayer-modulated memory effects by space charge polarization in resistive switching based on CuSCN-nanopyramids/ZnO-nanorods p-n heterojunction. *Sci. Rep.* **5**, 17859; doi: 10.1038/srep17859 (2015).
